# Frequent cross-resistance to rilpivirine among subtype C HIV-1 from first-line antiretroviral therapy failures in South Africa

**DOI:** 10.1177/2040206618762985

**Published:** 2018-03-22

**Authors:** Kerri J Penrose, Chanson J Brumme, Maritsa Scoulos-Hanson, Kristen Hamanishi, Kelley Gordon, Raquel V Viana, Carole L Wallis, P Richard Harrigan, John W Mellors, Urvi M Parikh

**Affiliations:** 1 Division of Infectious Diseases, Department of Medicine, University of Pittsburgh, Pittsburgh, Pennsylvania, USA; 2 Laboratory Program, 198129 British Columbia Centre for Excellence in HIV/AIDS, Vancouver, British Columbia, Canada; 3 Specialty Molecular Division, BARC-SA and Lancet Laboratories, Johannesburg, South Africa

**Keywords:** HIV, NNRTI, mutations, drug resistance

## Abstract

**Background:**

Rilpivirine (TMC278LA) is a promising drug for pre-exposure prophylaxis of HIV-1 because of its sub-nanomolar potency and long-acting formulation; however, increasing transmission of non-nucleoside reverse transcriptase inhibitor-resistant HIV-1 with potential cross-resistance to rilpivirine could reduce its preventive efficacy. This study investigated rilpivirine cross-resistance among recombinant subtype C HIV-1 derived from 100 individuals failing on first-line non-nucleoside reverse transcriptase inhibitor-containing antiretroviral therapy in South Africa whose samples were sent for routine HIV-1 drug resistance testing to Lancet Laboratories (Johannesburg, South Africa).

**Methods:**

Plasma samples were selected from individuals with HIV-1 RNA > 10,000 copies/ml and ≥1 non-nucleoside reverse transcriptase inhibitor-resistance mutation in reverse transcriptase. Recombinant HIV-1_LAI_-containing bulk-cloned full-length reverse transcriptase sequences from plasma were assayed for susceptibility to nevirapine (NVP), efavirenz (EFV) and rilpivirine in TZM-bl cells. Fold-change (FC) decreases in drug susceptibility were calculated against a mean IC_50_ from 12 subtype C HIV-1 samples from treatment-naïve individuals in South Africa. Cross-resistance was evaluated based on biological cutoffs established for rilpivirine (2.5-FC) and the effect of mutation combinations on rilpivirine phenotype.

**Results:**

Of the 100 samples from individuals on failing antiretroviral therapy, 69 had 2.5- to 75-fold decreased susceptibility to rilpivirine and 11 had >75-fold resistance. Rilpivirine resistance was strongly associated with K103N especially in combination with other rilpivirine-associated mutations.

**Conclusion:**

The frequently observed cross-resistance of HIV-1 suggests that the preventive efficacy of TMC278LA pre-exposure prophylaxis could be compromised by transmission of HIV-1 from individuals with failure of first-line non-nucleoside reverse transcriptase inhibitor-containing antiretroviral therapy.

## Introduction

Long-acting injectable antiretroviral agents are attractive as pre-exposure prophylaxis (PrEP) for HIV-1 prevention due to their requirement for infrequent dosing. TMC278LA, the long-acting formulation of the non-nucleoside reverse transcriptase inhibitor (NNRTI) rilpivirine (RPV), has a half-life of 91 days and a sub-nanomolar EC_50_ to wild-type HIV-1.^1,2^ TMC278LA administered at a dose of up to 1200 mg via intramuscular injection every four to eight weeks has been found to be safe and well tolerated in several Phase I and II clinical trials.^3–5^ Mathematical modeling predicts that TMC278LA has the potential to reduce new HIV-1 infections by up to 17% and be cost-effective if implemented in targeted high-risk populations.^6,7^

A major concern about TMC278LA is the potential risk of selection of NNRTI-resistant HIV-1 in newly infected persons because of the prolonged sub-therapeutic levels of TMC278LA after dosing is delayed or stopped.^8^ For example, in an RT-SHIV-infected macaque intravenously administered TMC278LA, the RPV-associated mutation E138Q was selected 17 weeks after the second TMC278 injection and conferred low level (4-fold) RPV resistance.^9^ In the Phase I SSAT040 trial, one participant was exposed to HIV-1 approximately 41 days after receiving a 300 mg dose of TMC278LA. Plasma RPV concentration was under 25 ng/µL when infection occurred. This was low enough to allow breakthrough infection of wild-type virus but high enough to subsequently select NNRTI resistance with K101E. RPV remained detectable in plasma below 50 ng/µL more than 200 days post-injection.^10^ Sub-therapeutic RPV levels in follow-up samples from the MWRI-01 study also persisted more than 500 days after a single-dose injection of 600 or 1200 mg TMC278LA.^11^

A second concern is whether RPV concentrations in the genital tract will be sufficient to protect against infection with either wild-type or NNRTI-resistant variants, the latter which are increasingly prevalent. Nearly six times more RPV was needed to attain EC_90_ in ectocervical tissues cultured with HIV-1_BaL_ compared to colonic tissue.^12^ In explant tissues from participants in MWRI-01 given a single 600 or 1200 mg dose of TMC278LA, HIV-1 was suppressed up to four months post-dosing in rectal tissue cultures, but was not suppressed in cervical and vaginal tissue cultures in the same time period.^5^ Sub-optimal drug levels in the genital tract could further increase the risk of infection by HIV-1, in particular by NNRTI-resistant variants.

Several studies have assessed the frequency of RPV cross-resistance against other NNRTI in the context of second- or third-line ART. In a genotypic evaluation of individuals experiencing virologic failure on NNRTI-based therapies that did not include RPV, 20% of sequences were found to contain mutations known to reduce susceptibility to RPV.^13^ Fourteen of 38 (37%) clinically derived HIV-1 subtype C clones having one or more NNRTI resistance mutation also demonstrated reduced susceptibility to RPV. This resistance was driven by the number of NNRTI resistance mutations per clone.^14^ In vitro analyses of NNRTI resistance mutations in both subtype B and C HIV-1 laboratory variants showed that >20-fold resistance to RPV was achieved by the NNRTI mutations Y181I/V and/or K101P.^14,15^ HIV-1_LAI_ with different, single NNRTI mutants maintained susceptibility to RPV, except for HIV-1 with Y181I and the double mutant K101P/V179I.^16^ No studies to date have examined RPV resistance arising from first-line ART.

The current study evaluated the activity of RPV against drug-resistant clinically derived subtype C HIV-1 arising from failure of first-line NNRTI-containing regimens from South Africa to evaluate the possible risks and benefits of using TMC278LA as a PrEP agent in regions where NNRTI-resistant subtype C HIV-1 may be circulating as a potentially transmissible variant.

## Methods

### Clinical samples

The sample set used in this analysis has been described previously.^17^ Plasma from 100 HIV-1 subtype C-infected individuals whose samples were sent for routine HIV-1 drug resistance testing to Lancet Laboratories (Johannesburg, South Africa) were evaluated. The specimens were from individuals who experienced virologic failure defined as having >10,000 HIV-1 RNA copies/ml after six months of nevirapine (NVP)-containing ART (N = 12 individuals) or efavirenz (EFV)-containing ART (N = 88 individuals; one individual had also taken single-dose NVP to prevent perinatal transmission). The viral population genotype of each sample had at least one major NNRTI resistance mutation in HIV-1 reverse transcriptase (RT) as defined by Stanford HIVdb v7.0.^18,19^ Plasma samples contained a median of 3 [Q1–Q3: 2–4] NNRTI-associated drug resistance mutations which included A98G, L100I, K101E/H, K103N/S, V106M, V108I, E138A/K, V179D/E Y181C, Y188L/C, G190A, H221Y, P225H, F227L, and M230L. The most frequent EFV and NVP resistance mutations were K103N in 55 of 100 samples (55%), V106M (44%) and G190A (26%). In total, 94 of 100 samples from patients failing ARV treatment also carried HIV-1 NRTI resistane mutations including M184V (82%), K65R (35%), L74I (19%), M41L (17%) or D67N (17%). Resistance mutations in the connection and RNAse H domains of RT spanning amino acids 320–560 included Y318F (3%) and N348I (14%). Additional subtype C resistance-associated C-terminal mutations/polymorphisms observed were G335D (86%), A371V (19%) and A376S (12%). From the treatment-naïve group, eight individuals had G335D and one individual had A376S.

Subtype C samples from 12 HIV-1 infected treatment-naïve individuals from the same geographical location were collected as controls. No NRTI or NNRTI resistance mutations were observed in any of the control sequences. All donor samples were anonymized, and testing was approved by the South African Medical Association Research Ethics Committee (SAMAREC) and the Institutional Review Board of the University of Pittsburgh.

### Generation of full-length patient-derived HIV-1xxLAI

Full-length patient-derived virus was generated as previously described.^17^ Patient-derived full-length RT was bulk cloned into xxLAIxhoI to preserve sequence diversity using the In-Fusion® HD Cloning System (Clonetech), and plasmid DNA was purified using the PureYield™ Plasmid Midiprep System (Promega). Lipofectamine2000 (Life Technologies) transfection into 293T cells was performed to generate infectious viral clones.

### HIV-1 phenotyping

A normalized input of 100 relative light units (RLU) was used to infect untreated or RPV-treated TZM-bl cells in a luciferase-based single-cycle drug susceptibility assay (Britelite Plus; Perkin–Elmer) as previously described.^9^ RPV is commercially available ARV for HIV treatment, and the long-acting version TMC278LA is an investigational product for HIV prevention, not yet approved by the FDA. RPV was obtained through the AIDS Research and Reference Reagent Program, Division of AIDS, NIAID, NIH: Rilpivirine (Cat# 12147) from Tibotec Pharmaceuticals, Inc. Four-parameter, non-linear regression for curve fitting was used to generate IC_50_ values using GraphPad Prism 6 software (GraphPad Software, Inc). All IC_50_s were evaluated as fold-change (FC) values determined by dividing the IC_50_ generated for each recombinant plasma-derived virus by the mean IC_50_ from duplicate determinations of the 12 treatment naïve plasma-derived subtype C viruses collected from the same geographical region against RPV. IC_90_ was estimated as double the IC_50_. The conventional biological cutoff (BCO) of 2.5-fold was used to define RPV resistance.^20,21^ This corresponds to 4 standard deviations above the mean treatment-naïve IC_50_ of 0.12 ng/mL as determined in this study. To address the effects of protein binding that occurs with human serum, a factor of 18.5^1^ was applied to all IC_50_ values obtained from cell culture.

### HIV-1 population genotyping

A laboratory-developed population genotyping assay was used to sequence HIV-1 from donor plasma at Lancet Laboratories, South Africa, as previously described.^22^ Full-length HIV-1 RT from plasma-derived xxLAI viral stocks were also sequenced using six bidirectional primers spanning the entire length of RT. Drug resistance mutations and virus subtype were identified using HIVdb v7.0 (Stanford).^18,19^ Phylogenetic analysis using the Stanford Calibrated Population Resistance Tool v6.0^23^ was done to ensure that plasma-derived cloned virus sequence clustered with virus in the original plasma sample. Overall, the sequences were 99% identical with a median hamming distance of 5/691 (1%). An ambiguity index was calculated as the percentage of ambiguous base calls (R, Y, K, M, S, W, B, D, H, V or N) in each sequence over the number of bases in the sequence.^24^ The median ambiguity index for plasma-derived virus (0.0072) was not significantly different from the median ambiguity index for recombinant virus (0.0087) (p > 0.05).

### Statistical analysis

Statistical analysis was conducted as previously described.^17^ Fisher’s exact test (FET) was used to assess differences in the prevalence of amino acids at all RT codons between samples with >2.5-FC RPV resistance and samples with lower resistance, including those from 12 treatment-naïve individuals (FC ≤ 2.5). A sensitivity analysis was performed by varying the FC cutoff at which RPV resistance was defined. Cutoffs of FC >5, >10 and >76 (the upper limit of the phenotypic resistance assay) were used. The additive effect of individual NNRTI resistance mutations (as defined by Stanford HIVdb v7.0)^18,19^ was investigated using FET. For each pairwise combination of NNRTI resistance-associated positions, the frequency of samples containing mutations at two codons and at a single codon was compared across resistance groups. A mutation was included in the analysis if it was present at an estimated >25% frequency within a sample as determined by population sequencing and if there were at least three observations of the mutation in the entire study data set. Correction for multiple comparisons was performed by controlling for the false discovery rate (FDR) using the method of Benjamini and Hochberg.^25^ An FDR-adjusted p value (or “q value”) of <0.15 was considered significant. Statistical analyses were performed in R (v3.1.2) with the glmnet library.

## Results

### Cross-resistance to rilpivirine of plasma-derived virus from individuals on failing first-line antiretroviral therapy

**Figure 1.**
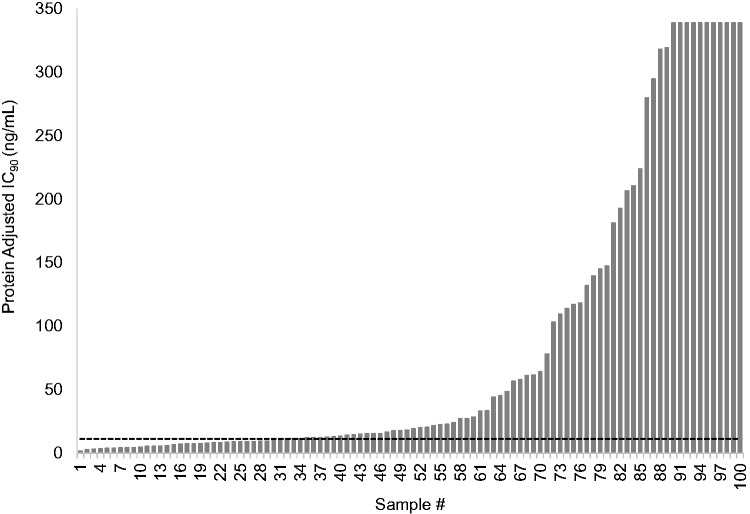
Rilpivirine Cross-Resistance of HIV-1 from 100 Individuals Failing First-Line Antiretroviral Therapy. The adjusted IC_90_ was calculated by multiplying the IC_50_ by 2 to approximate IC_90_ values and applying a rilpivirine (RPV) human serum binding factor (18.5) as determined in rilpivirine susceptibility experiments comparing human serum and fetal bovine serum (data not shown). RPV FC values were determined by dividing the IC_90_ generated for each plasma-derived virus by a composite IC_90_ from 12 treatment-naive plasma-derived viruses collected form the same geographical region. The dashed line indicates the RPV phenotypic biological cutoff of 2.5 FC. The IC_90_ values of samples 90 through 100 exceeded the highest concentration of RPV that could be tested in TZMbl cells without cytotoxicity and are reported as >340 ng/mL.

Plasma-derived recombinant viruses from 12 treatment-naive individuals yielded a mean RPV 50% inhibitory concentration (IC_50_) of 0.12 ± 0.04 ng/ml that was used as the wild-type, RPV-susceptible control value. Sixty-nine of 100 (69%) recombinant plasma-derived viruses containing full-length RT from individuals on failing first-line ART exceeded the BCO of 2.5 FC for RPV (Figure 1). Of those 69, 11 exceeded the upper limit of the assay (>IC_50_ of 9.2 ng/mL or >76 FC compared to the wild type). The median IC_50_ for the remaining 58 resistant viruses was 0.83 [Q1–Q3: 0.4–3.2] ng/mL or FC 7.0 [Q1–Q3: 3.6–26.4]. Thirty-one percent of specimens with NNRTI-associated drug resistance mutations were susceptible to RPV (FC < 2.5) (Table 1) (Supplementary Table S1). Protein-adjusted IC_90_ values are also presented in Figure 1 and Table 1.

**Table 1.** Cross-Resistance to Rilpivirine of Plasma-Derived Viruses from Individuals on Failing First-Line NNRTI-Based ART.

Resistance category	No. of samples (N = 100)	Median IC_50_ (Q1–Q3), ng/mL	Adjusted Median IC_90_ (Q1–Q3), ng/mL	Median fold-change (Q1–Q3)^a^
>76-fold^b^	11	>9.2	>340	>76
2.5-–76-fold	58	0.83 (0.4–3.1)	31 (16–116)	7.0 (3.6–26)
<2.5-fold	31	0.20 (0.12–0.24)	7.2 (7.2–8.8)	1.6 (1.0–2.0)

a Calculated against a mean IC_50_ from 12 patient-derived HIV-1_subtypeC_ viruses from South Africa.

b Rilpivirine concentrations > 9.2 ng/mL could not be tested due to toxicity.

### Prevalence of NNRTI resistance mutations associated with RPV cross-resistance

Sixteen NNRTI drug resistance mutations were analyzed individually to determine if specific mutations were associated with decreased susceptibility to RPV. After adjustment for multiple comparison, K103N was the only mutation associated with significant cross-resistance to RPV from individuals on failing NNRTI therapy at the q < 0.15 cutoff (p < 0.001) (Table 2). However, four out of four samples with K103N as the only NNRTI mutation were susceptible to RPV (FC range 0.9 to 2.0), indicating that additional resistance mutations were necessary for the reduction in RPV susceptibility conferred by K103N (Supplementary Table S1).

**Table 2.** Association of NNRTI resistance mutations with rilpivirine cross-resistance.

Mutation^a^	Resistant (≥2.5-fold)	Susceptible^b^ (<2.5-fold)			
	n = 69	n = 43	Odds ratio	p	q
NVP- and/or EFV-associated resistance mutations					
**K103N**	**44 (64%)**	**11 (26%)**	**5.12**	**<0.001**	**0.046**
V106M	22 (32%)	22 (51%)	0.44	0.049	0.84
V108I	13 (19%)	0 (0%)	Inf	0.002	0.167
P225H	11 (16%)	2 (5%)	3.88	0.199	1
RPV-associated resistance mutations					
K101E	8 (12%)	2 (5%)	2.69	0.312	1
E138A	7 (6%)	2 (5%)	2.31	0.478	1
E138K	3 (4%)	0 (0%)	Inf	0.284	1
V179I	8 (12%)	1 (2%)	5.51	0.150	1
V179L	0 (0%)	0 (0%)	na	na	na
H221Y	6 (9%)	2 (5%)	1.95	0.708	1
F227C	0 (0%)	0 (0%)	na	na	na
NVP-, EFV- and RPV-associated resistance mutations					
L100I	13 (19%)	0 (0%)	Inf	0.002	0.167
Y181C	15 (22%)	1 (2%)	11.67	0.004	0.276
Y188L	6 (9%)	0 (0%)	Inf	0.080	0.943
G190A	14 (20%)	12 (28%)	0.66	0.367	1
M230L	10 (14%)	1 (2%)	7.12	0.049	0.840

a Mutations listed are considered major NVP-, EFV- and/or RPV-associated as reported by Stanford HIV Drug Resistance Database and IAS 2017.^19,26^

b Sample size includes N = 12 “control” samples derived from ARV treatment naïve individuals from South Africa.Statistical significance is indicated with bold-faced text.

L100I, V108I and Y181C also showed a trend toward increased cross-resistance, but when adjusted for multiple comparisons, these were no longer significant. No other RPV-associated mutations in this sample set including K101E, E138A/K, V179I/L, Y188L, G190A, M230L, H221Y or F227C were associated with RPV cross-resistance (Table 2). No single mutation was associated with resistance in the >76 FC category.

### Combined effects of NNRTI resistance mutations on RPV cross-resistance

**Figure 2.**
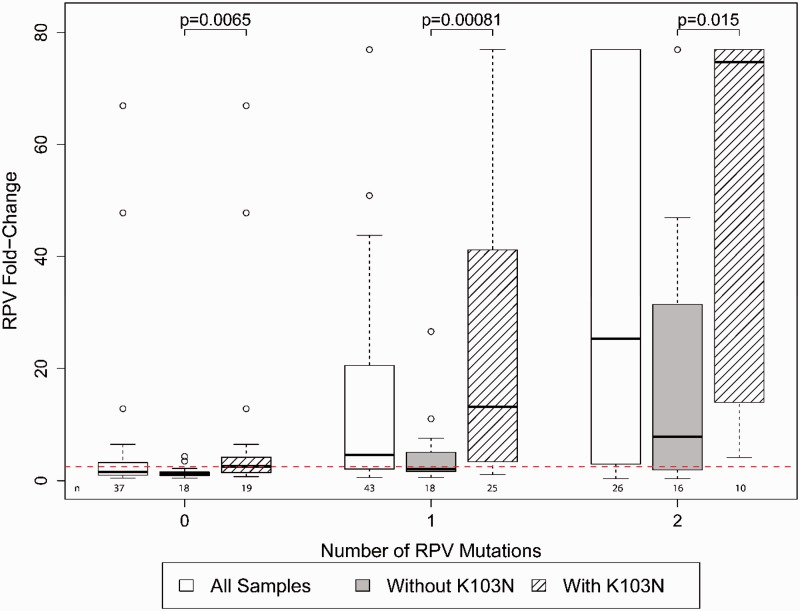
**Combined effect of NNRTI resistance mutations on RPV cross-resistance.** FC resistance was evaluated based on the contribution of the number of RPV-associated mutations (L100I, K101E/P, E138A/G/K/Q/R, V179L, Y181C/I/V, Y188L, H221Y, F227C and M230I/L) per sample with and without K103N. Samples are grouped based on having zero, one or two RPV-associated mutations. Samples containing three or more RPV-associated mutations could not be evaluated due to insufficient numbers. Samples with zero RPV-associated mutations include the 12 treatment-naïve individuals that were used as wild-type comparators in this study. Each group displays box-and-whisker plots of interquartile FC ranges for all samples in the category (white), samples without K103N (shaded) and samples containing K103N (diagonal stripes). The dotted red line indicates the RPV phenotypic resistance biological cutoff of 2.5 FC. RPV: rilpivirine.

The combination of K103N with other NNRTI resistance mutations was explored for its role in conferring cross-resistance to RPV. Fifty-five of 100 samples had HIV-1 genotypes that included K103N. As expected, the 19 of 55 (34%) samples with K103N and no RPV-associated mutations had relatively low FC values (median 2.6, IQR 1.4–4.2). By contrast, K103N in combination with one or two other RPV-associated mutations (including mutations at codons 100, 101, 138, 179, 181, 188, 221, 227 and/or 230)^26^ had a significantly higher median FC than samples with RPV-associated mutations without K103N (p ≤ 0.01). The number of RPV mutations per sample was associated with increased RPV resistance (p < 0.05) and the addition of K103N significantly enhanced the FC (p < 0.001) (Figure 2). In the absence of RPV mutations, a similar trend of higher levels of FC is observed with the addition of K103N with two or more NNRTI mutations, but the numbers were too small to achieve statistical significance.

## Discussion

Injectable monthly PrEP formulations could address the challenge of maintaining consistent drug levels over long periods of time to provide continuous protection against HIV-1 infection. This protection, however, could be compromised by exposure to drug-resistant HIV-1 from a partner failing ART, particularly when drug levels in the uninfected partner fall to suboptimal levels. In a recent survey in South Africa of newly diagnosed HIV-1 infections, transmitted NNRTI resistance occurred in 25 of 277 patients (9%) with ≥1 drug resistance mutations.^27^ Transmitted drug resistance is on the rise in South Africa and largely from NNRTI resistance.^28,29^ To evaluate the risk of breakthrough infection with TMC278LA by transmitted drug-resistant HIV-1, we evaluated the cross-resistance of plasma-derived viruses from individuals on failing NNRTI-based regimens to RPV, the active NNRTI in the long-acting formulation TMC278LA.

Overall, our study found that 69% of samples collected from individuals on a failing NNRTI-based ART displayed phenotypic RPV resistance above the BCO (Figure 1), causing concern for the use of TMC278LA as PrEP. The *c_min_* following successive 1200/600/600mg TMC0278LA monthly injections is 42.6ng/mL (Investigator’s Brochure) and the protein-adjusted IC_90_ values of 38% of the samples in our study would be projected to exceed the *c_min_* from this dosing strategy (Figure 1). In addition, the long “tail” of detectable RPV following the last dose of TMC278LA could promote selection of drug resistance in newly infected individuals as seen in the breakthrough infection in the SSAT040 trial.^10^ As a caveat, it is currently unknown if the drug that is detected during the prolonged tail of RPV is bioavailable and active or is protein-bound and incapable of conferring drug pressure for resistance selection.

The most frequently transmitted NNRTI mutation is K103N,^30^ but relatively little is known clinically about cross-resistance of K103N to RPV. In a small study of three patients who had HIV-1 with K103N from prior NNRTI-containing therapy with no other NNRTI mutations, all three responded well to a switch from boosted protease inhibitor-based second-line therapies to RPV/tenofovir/emtricitabine.^31^ In the ECHO and THRIVE trials, pre-existing K103N alone did not reduce RPV susceptibility in participants who added RPV to an existing NRTI-based ART regimen.^32^ In our study, K103N was found in 55 of 100 samples collected from an ART-experienced study population. Additionally, of 69 samples with ≥ 2.5-fold decrease in susceptibility to RPV, 44 (64%) had K103N. The decrease in susceptibility was due to the additive effect of K103N with other RPV-associated NNRTI resistance mutations (Figure 2). Other studies have reported an association of RPV resistance with K103N combined with other NNRTI resistance mutations, especially L100I, in clinical samples.^33–35^ The clinical implications of the interactions between K103N and NNRTI mutations in conferring RPV resistance is unknown, and should be monitored as the use of RPV expands for both treatment and prevention.

In summary, this study showed that cross-resistance to RPV is common for NNRTI-resistant viruses from individuals experiencing virologic failure while on a first-line regimen and could reduce the efficacy of TMC278LA for HIV-1 prevention. The highest risk of breakthrough infection is likely to occur during the long RPV tail following discontinuation of TMC278LA dosing. The long tail may also be when selection of RPV-resistant HIV-1 is most likely to occur in newly infected individuals. Assessing the preventive efficacy of TMC278LA and monitoring resistance in TMC278LA users will be critical to better understand its role for PrEP.

## Supplemental Material

Supplementary table - Supplemental material for Frequent cross-resistance to rilpivirine among subtype C HIV-1 from first-line antiretroviral therapy failures in South Africa Click here for additional data file. Supplementary Material

## References

[1] Azijn H Tirry I Vingerhoets J et al TMC278, a next-generation nonnucleoside reverse transcriptase inhibitor (NNRTI), active against wild-type and NNRTI-resistant HIV-1. Antimicrob Agents Chemother 2010; 54: 718–727. 19933797 10.1128/AAC.00986-09 PMC2812151

[2] Janssen PA Lewi PJ Arnold E et al In search of a novel anti-HIV drug: multidisciplinary coordination in the discovery of 4-[[4-[[4-[(1E)-2-cyanoethenyl]-2,6-dimethylphenyl]amino]-2-pyrimidinyl]amino]benzonitrile (R278474, rilpivirine). J Med Chem 2005; 48: 1901–1909. 15771434 10.1021/jm040840e

[3] Bekker L-G Li SS Tolley B et al. HPTN 076: TMC278 LA safe, tolerable, and acceptable for HIV preexposure prophylaxis. In: *Conference on retroviruses and opportunistic infections*, 13–16 February 2017, Seattle, WA, abstract 421LB.

[4] Verloes R Deleu S Niemeijer N et al Safety, tolerability and pharmacokinetics of rilpivirine following administration of a long-acting formulation in healthy volunteers. HIV Med 2015; 16: 477–484. 25988676 10.1111/hiv.12247

[5] McGowan I Dezzutti CS Siegel A et al Long-acting rilpivirine as potential pre-exposure prophylaxis for HIV-1 prevention (the MWRI-01 study): an open-label, phase 1, compartmental, pharmacokinetic and pharmacodynamic assessment. Lancet HIV 2016; 3: e569–e578. 27658864 10.1016/S2352-3018(16)30113-8

[6] Glaubius RL Parikh UM Hood G et al Deciphering the effects of injectable pre-exposure prophylaxis for combination human immunodeficiency virus prevention. Open Forum Infect Dis 2016; 3: ofw125 27703992 10.1093/ofid/ofw125 PMC5047428

[7] Glaubius RL Hood G Penrose KJ et al Cost-effectiveness of injectable preexposure prophylaxis for HIV prevention in South Africa. Clin Infect Dis 2016; 63: 539–547. 27193745 10.1093/cid/ciw321 PMC4967601

[8] Landovitz RJ Kofron R andMcCauley M. The promise and pitfalls of long-acting injectable agents for HIV prevention. Curr Opin HIV AIDS 2016; 11: 122–128. 26633643 10.1097/COH.0000000000000219 PMC4747082

[9] Melody K McBeth S Kline C et al Low frequency of drug-resistant variants selected by long-acting rilpivirine in macaques infected with simian immunodeficiency virus containing HIV-1 reverse transcriptase. Antimicrob Agents Chemother 2015; 59: 7762–7770. 26438501 10.1128/AAC.01937-15 PMC4649225

[10] Penrose KJ Parikh UM Hamanishi KA et al Selection of rilpivirine-resistant HIV-1 in a seroconverter from the SSAT 040 trial who received the 300-mg dose of long-acting rilpivirine (TMC278LA). J Infect Dis 2016; 213: 1013–1017. 26563240 10.1093/infdis/jiv528

[11] McGowan I Siegel A Engstrom J et al. Persistence of rilpivirine following single dose of long-acting injection. In: *21st international AIDS conference*, 18–22 July 2016, Durban, South Africa, abstract TUACO 103.

[12] Dezzutti CS Else LJ Yandura SE et al Distinct pharmacodynamic activity of rilpivirine in ectocervical and colonic explant tissue. Antimicrob Agents Chemother 2016; 60: 2765–2770. 26902757 10.1128/AAC.00167-16 PMC4862523

[13] Anta L Llibre JM Poveda E et al Rilpivirine resistance mutations in HIV patients failing non-nucleoside reverse transcriptase inhibitor-based therapies. AIDS 2013; 27: 81–85. 22842995 10.1097/QAD.0b013e3283584500

[14] Basson AE Rhee SY Parry CM et al Impact of drug resistance-associated amino acid changes in HIV-1 subtype C on susceptibility to newer nonnucleoside reverse transcriptase inhibitors. Antimicrob Agents Chemother 2015; 59: 960–971. 25421485 10.1128/AAC.04215-14 PMC4335849

[15] Giacobbi NS andSluis-Cremer N. In vitro cross-resistance profiles of rilpivirine, dapivirine, and MIV-150, nonnucleoside reverse transcriptase inhibitor microbicides in clinical development for the prevention of HIV-1 infection. Antimicrob Agents Chemother 2017; 61: pii: e00277-17. 10.1128/AAC.00277-17 PMC5487620 28507107

[16] Smith SJ Pauly GT Akram A et al Rilpivirine and doravirine have complementary efficacies against NNRTI-resistant HIV-1 mutants. J Acquir Immune Defic Syndr 2016; 72: 485–491. 27124362 10.1097/QAI.0000000000001031 PMC4942337

[17] Penrose KJ Wallis CL Brumme CJ et al Frequent cross-resistance to dapivirine in HIV-1 subtype C-infected individuals after first-line antiretroviral therapy failure in South Africa. Antimicrob Agents Chemother 2017; 61: pii: e01805-16. 10.1128/AAC.01805-16 PMC5278732 27895013

[18] Paredes R Tzou PL van Zyl G et al Collaborative update of a rule-based expert system for HIV-1 genotypic resistance test interpretation. PLoS One 2017; 12: e0181357 28753637 10.1371/journal.pone.0181357 PMC5533429

[19] Rhee SY Gonzales MJ Kantor R et al Human immunodeficiency virus reverse transcriptase and protease sequence database. Nucleic Acids Res 2003; 31: 298–303. 12520007 10.1093/nar/gkg100 PMC165547

[20] AIDSinfo. Rilpivirine, FDA-label. United States Department of Health and Human Services, https://aidsinfo.nih.gov/drugs/426/rilpivirine/0/professional (2017, accessed 19 February 2017).

[21] Monogram Bioschences. Phenosense HIV drug resistance assay, https://www.monogrambio.com/sites/monogrambio/files/imce/uploads/PS_report_new_Watermark.pdf08/18/2017 (2017, accessed 19 February 2017).

[22] Wallis CL Papathanasopoulos MA Lakhi S et al Affordable in-house antiretroviral drug resistance assay with good performance in non-subtype B HIV-1. J Virol Meth 2010; 163: 505–508. 10.1016/j.jviromet.2009.11.011 PMC2932961 19917318

[23] Gifford RJ Liu TF Rhee SY et al The calibrated population resistance tool: standardized genotypic estimation of transmitted HIV-1 drug resistance. Bioinformatics 2009; 25: 1197–1198. 19304876 10.1093/bioinformatics/btp134 PMC2672634

[24] Andersson E Shao W Bontell I et al Evaluation of sequence ambiguities of the HIV-1 pol gene as a method to identify recent HIV-1 infection in transmitted drug resistance surveys. Infect Genet Evol J Mol Epidemiol Evolut Genet Infect Dis 2013; 18: 125–131. 10.1016/j.meegid.2013.03.050 PMC4066879 23583545

[25] Benjamini Y andYosef H. Controlling the false discovery rate: a practical and powerful approach to multiple testing. J R Stat Soc Ser B 1995; 57: 289–300.

[26] Wensing AM Calvez V Gunthard HF et al 2017 Update of the drug resistance mutations in HIV-1. Top Antivir Med 2017; 24: 132–133. 28208121 PMC5677049

[27] Steegen K Carmona S Bronze M et al Moderate levels of pre-treatment HIV-1 antiretroviral drug resistance detected in the first South African National Survey. PLoS One 2016; 11: e0166305. 27907009 10.1371/journal.pone.0166305 PMC5132262

[28] Manasa J Danaviah S Lessells R et al Increasing HIV-1 drug resistance between 2010 and 2012 in adults participating in population-based HIV surveillance in rural KwaZulu-Natal, South Africa. AIDS Res Hum Retroviruses 2016; 32: 763–769. 27002368 10.1089/aid.2015.0225 PMC4971422

[29] Rhee SY Blanco JL Jordan MR et al Geographic and temporal trends in the molecular epidemiology and genetic mechanisms of transmitted HIV-1 drug resistance: an individual-patient- and sequence-level meta-analysis. PLoS Med 2015; 12: e1001810. 25849352 10.1371/journal.pmed.1001810 PMC4388826

[30] Rhee SY Jordan MR Raizes E et al HIV-1 drug resistance mutations: potential applications for point-of-care genotypic resistance testing. PLoS One 2015; 10: e0145772. 26717411 10.1371/journal.pone.0145772 PMC4696791

[31] Rokx C Verbon A andRijnders BJ. Successful switch to rilpivirine/tenofovir/emtricitabine in HIV-1-infected patients with an isolated K103N mutation acquired during prior nonnucleoside reverse transcriptase inhibitor therapy. HIV Med 2014; 15: 611–614. 24738660 10.1111/hiv.12157

[32] Vingerhoets J Rimsky L Van Eygen V et al Pre-existing mutations in the rilpivirine Phase III trials ECHO and THRIVE: prevalence and impact on virological response. Antivir Ther 2013; 18: 253–256. 22951490 10.3851/IMP2358

[33] Picchio GR Rimsky LT Van Eygen V et al Prevalence in the USA of rilpivirine resistance-associated mutations in clinical samples and effects on phenotypic susceptibility to rilpivirine and etravirine. Antivir Ther 2014; 19: 819–823. 24704709 10.3851/IMP2771

[34] Haddad M Napolitano LA Frantzell A et al. Combinations of HIV-1 reverse transcriptase mutations L100I ± K103N/S and L100I ± K103R ± V179D reduce susceptibility to rilpivine. In: *Interscience conference on antimicrobial agents and chemotherapy* (ICAAC), Denver, CO, 2013, abstract H-677.

[35] Theys K Camacho RJ Gomes P et al Predicted residual activity of rilpivirine in HIV-1 infected patients failing therapy including NNRTIs efavirenz or nevirapine. Clin Microbiol Infect 2015; 21: 607.e1–607.e8. 10.1016/j.cmi.2015.02.011 25704446

