# Supplementary material for: Frequent cross-resistance to rilpivirine among subtype C HIV-1 from first-line antiretroviral therapy failures in South Africa
Source: Antivir Chem Chemother. 2018 Mar 22;26:2040206618762985. doi: 10.1177/2040206618762985 (PMC5890541; doi:10.1177/2040206618762985)
Supplement: Supplementary table - Supplemental material for Frequent cross-resistance to rilpivirine among subtype C HIV-1 from first-line antiretroviral therapy failures in South Africa [file Supplementary_table.pdf]

Supplementary Table S1. Rilpivirine IC_{50}, Fold-Change and Resistance Category, and Reverse Transcriptase Genotype for Each Plasma-Derived HIV-1 from Individuals on Failing First-Line Non-Nucleoside Reverse Transcriptase Inhibitor-Based Antiretroviral Therapy

Sample	FC[a]	IC_{50} (ng/mL)	Protein Adjusted IC_{90}	Major NRTI[b]	Major NNRTI[b]
1	0.36	0.04	1.6	A62AV,L74IL,M184V	K101E,V106M,E138A,G190A
2	0.58	0.07	2.6	D67N,K70R,M184V,T215Y,K219Q	V106M,F227L,N348I
3	0.74	0.09	3.3	K65R,L74I,Y115F,M184V	K103N,V106M
4	0.76	0.09	3.3	D67N, K70E, M184V	V106M, G190A, H221Y, F227L
5	0.84	0.10	3.7	None	V106MV,G190AG
6	0.87	0.10	3.8	None	K103N
7	0.95	0.11	4.2	K70E,L74IL,Y115F,M184V	A98G,V106M,Y188C
8	1.0	0.12	4.3	None	K103N
9	1.0	0.12	4.4	T69NT	K103N
10	1.1	0.13	4.8	M41L,D67N,V75I,M184V,L210W,T215Y	K103N,V106M,F227FL
11	1.2	0.14	5.3	V75M,M184V	V106M,E138A,G190A
12	1.2	0.15	5.5	M41L,M184V,T215F	V106M,G190A
13	1.2	0.15	5.5	M184V	K103N,N348I
14	1.4	0.16	6.0	A62V,K65R,L74I,Y115F,M184V	V106M,Y188C,G190A
15	1.5	0.18	6.7	A62V,K65R,M184V	K103S,V106M
16	1.6	0.20	7.2	A62V,K65R,L74IL,M184V	K103N,G190A
17	1.6	0.20	7.3	M184V	V106M,G190A,H221Y
18	1.6	0.20	7.3	M184V	K101HKNQ,K103N,G190A
19	1.7	0.21	7.6	L74V,Y115F,M184V	K103Q,V106M,Y188F,F227L
20	1.8	0.22	8.0	K65R,M184V	V106M,Y188C
21	1.8	0.22	8.1	K65R,M184V	V106M,V179D
22	1.9	0.22	8.3	M41L,D67N,K70E,M184V,T215F	K101H,V106M,G190A,F227L
23	2.0	0.24	8.7	K65R,M184V	V106M,V179D,M230L
24	2.0	0.24	8.8	L74IL,Y115F,F116FY,M184V,K219EK	V106M,Y188C

25	2.0	0.24	8.9	L74I,M184V	K103N
26	2.1	0.25	9.1	D67N,T69N,K70R,L74IL,M184V,T215IV,K219E	K103N,P225H
27	2.1	0.25	9.1	K65R,D67G,Y115F,M184V,K219E	V106M,Y181C,G190A
28	2.1	0.25	9.2	M41L,D67N,K70R,L74I,M184V,T215Y,K219Q	K101E,G190A,P225H
29	2.2	0.26	9.6	L74IL,M184V	K103S,V106M
30	2.2	0.26	9.6	K65R,L74I,M184V,K219E	K103N,V106M
31	2.2	0.27	9.8	K65R,L74I,Y115F,M184V,K219E	V90I,V106M,E138G,V179E,F227L
32	2.5	0.30	11	M184V	K103N,N348I
33	2.6	0.31	11	M184V,T215F	A98G,K103N,V108I
34	2.6	0.31	12	D67N,K70R,M184V,K219Q	K103N,V106M,E138AEKT,N348I
35	2.7	0.32	12	M41LM,K65R,M184V	V106M,E138A,V179D
36	2.8	0.33	12	M41L,D67N,M184V,T215F,K219W	A98G,K101E,G190A
37	2.8	0.33	12	M184V	K103N,E138A,P225H
38	2.9	0.34	13	M184V	K103N,V106M,E138Q,N348IN
39	2.9	0.35	13	M41L,D67N,T69D,K70R,M184V,T215F,K219Q	K101H,V106M,G190A,F227L
40	3.1	0.37	14	None	K101EK,K103KN,V106M,V108IV,G190AG
41	3.2	0.38	14	T69N,K70R,M184V	V106M,V179D
42	3.3	0.39	14	D67N,K70G,M184V,K219Q	K103NS,V106M,G190A,N348I
43	3.4	0.40	15	A62AV,K65R,M184I	V90I,A98G,K103N,Y181C,G190A
44	3.5	0.41	15	M184MV	K103N,E138A,P225H
45	3.5	0.42	15	M41L,L74I,M184V,T215F	K103N,V108I
46	3.5	0.42	15	L74V,V75L,M184V,K219N	G190E
47	3.8	0.45	17	M41LM,M184V,T215Y	K103N,V108I
48	4.0	0.47	18	K65R,Y115F,M184V	L100M,K101EK,V106M,E138K,V179DV,F227L
49	4.0	0.48	18	K70E,Y115F,M184V	V106M,V179D
50	4.1	0.49	18	L74IL,M184V,T215NSTY	K101EK,K103N,V108IV,P225H,M230LM
51	4.3	0.52	19	L74V,Y115F,M184V	K103S,G190A,Y318F
52	4.6	0.54	20	K70EK,L74V,M184V	L100I,K103N,V106MV
53	4.6	0.55	20	K65R,L74IL,Y115F,M184V	K103N,P225H
54	4.9	0.59	22	A62AV,K65KR,K70EK,M184V	K103N,Y181C

55	5.1	0.61	22	M41LM,V75M,M184V,T215Y	A98G,V106M,F227L
56	5.2	0.61	23	M41L,D67N,T69D,K70R,M184V,T215F,K219Q	K103N,V106M,E138A,F227L
57	5.4	0.65	24	L74V,M184V	V179D,Y188L,N348IT
58	6.2	0.74	27	M184V	Y181C
59	6.2	0.74	27	None	K103N,E138EG,P225H
60	6.5	0.77	28	K219KR	K103N,P225H
61	7.5	0.89	33	M41L,K65R,T69V,K70T,M184V	K101E,V106M,Y181C,G190A,H221Y,F227L
62	7.6	0.90	33	M184V	Y181C,N348IT
63	10	1.2	44	K65R,L74IL,M184V	K103NS,V106MV,M230L
64	10	1.2	45	K65R	V106M,V179D,Y181C
65	11	1.3	49	V75M, F77L, M184V, T215Y	V106A, G190A, F227L
66	13	1.5	57	T69NT,K70KR,M184V	K103N,P225H,N348I
67	13	1.6	58	K65R,M184V	L100I,K103N
68	14	1.7	61	K70EK,M184V,T215FIST	V90IV,K103N,V108IV,Y181CY,G190AG,K238KT,N348IN
69	14	1.7	62	D67N, K70E, V75T, M184V	A98AG, K103N, V106IM, V108IV, Y181C, G190A, H221Y
70	15	1.7	64	A62AV,V75IT,M184I	K103N,P225HP,M230L
71	18	2.1	78	K65R,M184V	V106M,E138A,V179D,M230L
72	23	2.8	103	K65R,T69 deletion	K101E,K103KN,E138AT,Y181C,G190AT
73	25	3.0	109	A62V,K65R,M184I	V106M,V179D,M230L
74	26	3.1	114	M41L, V75I, M184V, T215Y	Y188L, H221Y, N348IN
75	27	3.2	117	M41L,T69AD,T215Y,K219KR	A98G,Y181C,G190S
76	27	3.2	118	M41L, D67N, T69D, M184I, L210W, T215Y	V90I, L100I, K103N
77	30	3.6	132	K65R,V75I,M184V,K219E	V90I,V108I,V179D,Y181C,G190A
78	32	3.8	140	K65R,M184V	L100I,K103N
79	33	3.9	145	K65R,K70KT,M184V	A98G,K101E,V106I,V108IV,Y181C,G190A
80	33	4.0	148	K65R,M184V	L100I,K103N
81	41	4.9	181	M184V,K219E	L100I,K103N
82	44	5.2	193	K65R,L74I,M184V	L100I,K103N
83	47	5.6	207	K70KN,L74V,Y115F,M184V	A98G,Y181C,H221Y,Y318F
84	48	5.7	211	M41L,D67N,K70KNRS,L74I,M184V,T215F,K219Q	A98G,K103N,P225H,K238N

85	51	6.1	224	K65R,K70KT,L74IL,M184V,K219KQ	K103NS,V106M,M230L
86	64	7.6	280	L74V,M184V	L100I,K103N,V108I,H221HY
87	67	8.0	295	D67N, T69D, K70R, M184V, T215V, K219Q	A98G, K103N, P225H
88	72	8.6	318	K65R, T69 deletion, K70KN, L74LV, Y115F, K219R	K101E, V106M, Y181C, G190A, H221Y
89	73	8.6	320	None	A98AG,K103N,V106MV,V179DINV,Y188FHLY
90	77	9.2	339	T69N,K70R,M184V,T215I,K219Q	A98G,K103N,V108I,M230L
91	77	9.2	339	K70E,M184V	L100I,K103N,V108IV,E138K,P225HP
92	77	9.2	339	D67G,M184V	L100IL,Y188L
93	77	9.2	339	M41L,D67N,K70KR,M184V,L210LW,T215Y,K219E	A98G,V179DV,Y188L
94	77	9.2	339	K65R,V75M,M184I	K103N,V106M,M230L
95	77	9.2	339	D67N,K70R,M184V,T215FI,K219E	K103N,V108I,V179E,N348I
96	77	9.2	339	K65R,T69 deletion	K103N,V179E,Y181C
97	77	9.2	339	K65R,K70KT,M184V	K103N,V179T,Y188L
98	77	9.2	339	K65R,L74V,M184V	L100I,K103N,M230L
99	77	9.2	339	A62AV,K65R,Y115FY,M184V	L100I,K103N
100	77	9.2	339	K65R,M184V,K219E	L100I,K103N,M230L,Y318F

[a] Fold-change resistance (FC) was calculated by dividing the IC_{50} generated for each patient derived virus by a composite IC_{50} from 12 treatment naïve patient derived viruses collected form the same geographical region.

[b] Genotypic information was obtained through HIVdb v7.0 (Stanford University). Major NRTI and NNRTI resistance mutations are reported directly as determined by HIVdb v7.0.
